# One-Pot Green Synthesis of Graphene Nanosheets Encapsulated Gold Nanoparticles for Sensitive and Selective Detection of Dopamine

**DOI:** 10.1038/srep41213

**Published:** 2017-01-27

**Authors:** Balamurugan Thirumalraj, Chellakannu Rajkumar, Shen-Ming Chen, Selvakumar Palanisamy

**Affiliations:** 1 Department of Chemical Engineering and Biotechnology, National Taipei University of Technology, Taipei 106, ROC, Taiwan

## Abstract

We report a simple new approach for green preparation of gallic acid supported reduced graphene oxide encapsulated gold nanoparticles (GA-RGO/AuNPs) via one-pot hydrothermal method. The as-prepared composites were successfully characterized by using Fourier transform infrared spectroscopy (FTIR), Raman spectroscopy, X-ray powder diffraction techniques (XRD), scanning electron microscope (SEM), high resolution transmission electron microscopy (HRTEM) and elemental analysis. The GA-RGO/AuNPs modified electrode behaves as a hybrid electrode material for sensitive and selective detection of dopamine (DA) in presence of ascorbic acid (AA) and uric acid (UA). The GA-RGO/AuNPs modified electrode displays an excellent electrocatalytic activity towards the oxidation of DA and exhibits a wide linear response range over the DA concentrations from 0.01–100.3 μM with a detection limit (LOD) of 2.6 nM based on *S/N = 3*. In addition, the proposed sensor could be applied for the determination of DA in human serum and urine samples for practical analysis.

Dopamine (DA, 3,4-dihydroxy phenylalanine) is an essential biological small molecule, which plays an important biological roles in human metabolism as well as central nervous system of mammalian^1^,^2^,^3^. The higher concentration of DA is a cardiotoxic including death of heart muscles, high blood pressure and high heart rate. Besides, the higher concentration of DA will cause a serious of neurological disorders such as Parkinson’s disease, hypertension, and schizophrenia^4^,^5^,^6^. Therefore, the low level detection of DA in biological samples has been much important in analytical and biomedical applications. To date, the electrochemical methods have been widely considered for the determination of DA owing to their remarkable properties such as simplicity, low cost, eco-friendly and higher sensitivity^2^. Over the past decays, various modified electrodes have been utilized for the determination of DA in the literature. However, these modified electrodes have not suitable for the determination of DA due to poor selectivity, sensing in higher oxidation potential and fouling to the signal response^7^,^8^. Hence, there is a demand to develop the modified electrodes with those requirements that can be employed by exploiting the hybrid nanomaterials.

So far, numerous hybrid nanomaterials have been constructed for the electrochemical sensor and biosensor applications. Specifically, reduced graphene oxide (RGO) is a well known sp^2^ hybridized 2D planar carbon nanosheets, have been received much attention for assembling different nanomaterials due to its structural, electrical and conducting properties^9^,^10^,^11^. However, the chemical modifications and/or functionalizations have been used frequently to improve their structural, physiochemical and conducting properties by introducing the new functional groups^12^,^13^,^14^. Meanwhile, the noble metal nanoparticles such as Pt, Au, and Ag have been attained much attention in catalysis owing to their unique physical and chemical properties^15^. In particular, gold nanoparticles (AuNPs) have been widely adapted in various potential applications such as electrocatalysis and biosensor due to their excellent conductivity, specific large surface area and good biocompatibility^16^,^17^,^18^. Gallic acid (GA, 3,4,5-trihydroxybenzoic acid) is a polyphenolic compound consists of three adjacent hydroxyl groups at benzene ring which facilitates considerable ability to reduce the GO^19^,^20^. Besides, the GA has been covalently attached with the edge plane of GR sheets through H-bonding between the OH groups of GA^21^,^22^. Here, GA acts as a functionalization and reducing as well as stabilizing agent for the preparation of GA-RGO/AuNPs. In previous reports, Stathi *et al*. reported the immobilization of gallic acid on graphene oxide for XPS and EPR Study with long term stability in both solid form and aqueous solution^21^. Li *et al*. studied the dispersion ability of reduced graphene oxide at room temperature using GA as a reducing and stabilizing agent with excellent dispersion ability both in water and organic solvents^20^. Xu *et al*. was prepared reduced graphene oxide by GO was deoxygenated with GA for Li-storage applications with satisfactory storage performance^23^. To the best of our knowledge, there are no reports available for the determination of DA using GA supported RGO/AuNPs.

Conversely, the analytical performance of the proposed sensor such as linear response range and detection limit (LOD) for DA has been compared with previously reported DA sensor using AuNPs and RGO nanohybrids. For example, Liu *et al*. used Layer-by-layer assembled multilayer films of reduced graphene oxide/gold nanoparticles for the electrochemical detection of dopamine. The sensor presented the response range is 1–60 μM with a detection limit of 20 nM based on *S/N* = 3^24^. Rao *et al*. utilized the glassy carbon electrode modified with MnO_2_, graphene oxide, carbon nanotubes and gold nanoparticles for DA sensor. The sensor showed the response range for DA is 0.5 μM to 2.5 mM with a detection limit of 0.17 μM^25^. Thanh *et al*. prepared the seed assisted synthesis of gold nanoparticle/nitrogen-doped graphene nanohybrids for electrochemical detection of glucose and DA. The sensor displayed the response range for DA is 38 nM to 48 μM with a detection limit of 10 nM^18^. Khudaish *et al*. fabricated the glassy carbon electrode modified with poly(2,4,6-triaminopyrmidine) decorated AuNPs for DA sensor. The sensor exhibited the response range for DA is 0.15–1.5 μM with a detection limit of 17 nM^26^. Baig *et al*. prepared the direct electrochemical reduced graphene oxide and AuNPs for simultaneous detection of DA and uric acid which shows the response range for DA is 0.1–25 μM with a detection limit of 24 nM^27^. Kwak *et al*. used thermally reduced AuNPs/graphene nanocomposite for electrochemical detection of DA. The sensor showed the response range for DA is 0.1–100 μM with a detection limit of 95 nM^28^. Hou *et al*. used the hierarchical nanoporous AuAg alloy for the sensitive detection of DA and uric acid. The sensor displayed the response range for DA is 5–335 μM with a detection limit of 0.2 μM^29^. Vinoth *et al*. prepared AuNPs/RGO nanocomposite using a facile “single-step one-pot approach” for electrochemical detection of DA and uric acid. The sensor exhibited the response range for DA is 0.05–11 μM with a detection limit of 20 nM^30^. Among them, the advantage of our proposed sensor showed a much lower detection limit (LOD) and good linear response range towards the detection of DA.

In this study, we report a one-pot facile synthesis of GA-RGO/AuNPs composite for sensitive and selective electrochemical detection of DA for the first time. The functionalization of GA-RGO was achieved by the bond formation between the carboxylic group of graphite oxide and the phenolic group of GA. The as-prepared GA-RGO/AuNPs composite was highly stable on the electrode surface and possessed excellent electrocatalytic activity and good sensing performance towards the detection of DA. The GA-RGO/AuNPs modified electrode was successfully applied for the determination of DA *via* differential pulse voltammetry (DPV), with excellent analytical performance such as good linear response, lower detection limit (LOD) and better sensitivity. The sensor was achieved a good recovery for the determination of DA in human serum and urine samples. The overall procedure of the preparation of GA-RGO/AuNPs composite was shown in Fig. 1.

## Results and Discussion

### Material characterizations

**Figure 2:**
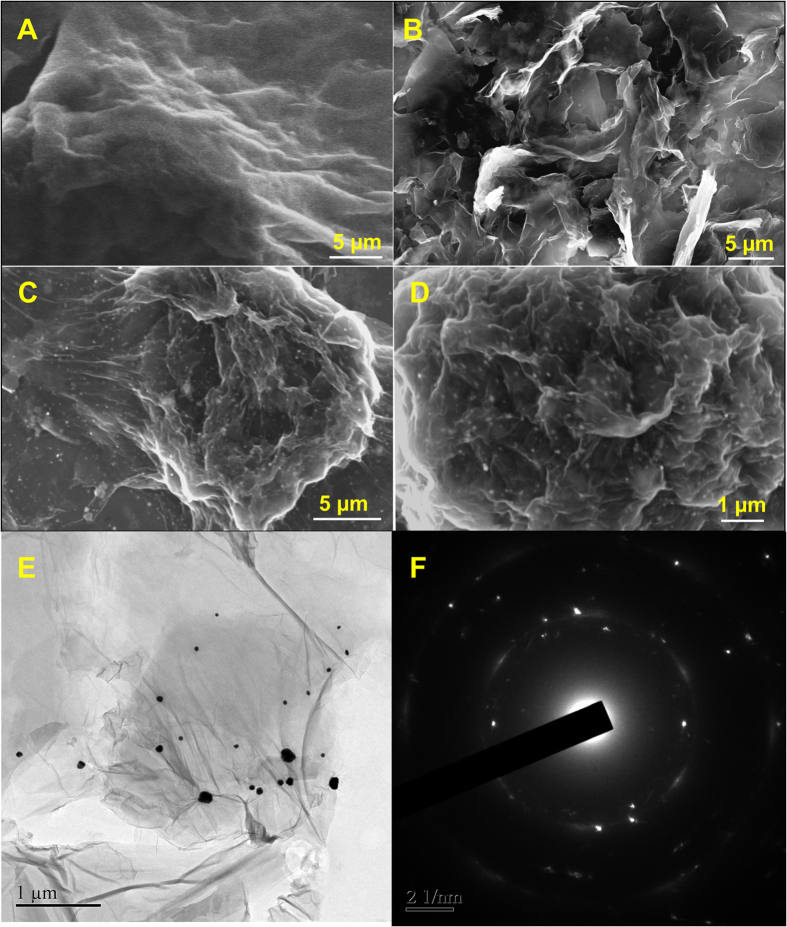
SEM images of (**A**) GO, (**B**) RGO, (**C**) lower and (**D**) higher magnification of GA-RGO/AuNPs. (**E**) HRTEM and (**F**) SAED pattern of GA-RGO/AuNPs composite.

The structural morphology of the as-prepared hybrid materials were characterized by SEM and TEM analysis. Figure 2 shows the SEM and TEM images of (A) GO, (B) RGO and (C) GA-RGO/AuNPs composites. As can be seen that the GO showed a crumpled and folded sheet like structures (Fig. 2A). After the hydrothermal reaction, these folded sheets of GO were well reduced and exfoliated sheet like morphology has been observed which leads to the better surface area (Fig. 2B). The similar kind of morphology has been observed in the literatures, suggesting the successful formation of RGO^31^,^32^.

The lower and higher magnified SEM images of GA-RGO/AuNPs (Fig. 2C and D), which suggest that the spherical shaped AuNPs has been observed on the surface of RGO nanosheets. These AuNPs were uniformly distributed on the surface of RGO nanosheets. In addition, a well-defined structural morphology of GA-RGO/AuNPs composite was further confirmed by TEM analysis, as shown in Fig. 2E. The TEM image of GA-RGO/AuNPs (Fig. 2E) composite revealed that a crystalline tiny AuNPs (with an average diameter of 43 ± 5 nm) were uniformly distributed on the surface of RGO nanosheets. The SAED pattern of GA-RGO/AuNPs clearly showed the AuNPs are decorated uniformly to form the crystalline nanoparticles, as shown in Fig. 2F. Furthermore, the elemental mapping and weight percentage of the composite also supports the conformation of GA-RGO/AuNPs, as shown in Fig. S1. As seen from these results, suggesting that the presence of carbon (C), oxygen (O), and gold (Au) elements in GA-RGO/AuNPs composite Fig. S1(B–D). The C and O elements originated from the graphene nanosheets and Au from AuNPs on the surface of graphene. The percentage of elemental composition C, O and Au were showed in Fig. S1A (inset). Based on these results, strongly suggests the successful formation of GA-RGO/AuNPs composite.

**Figure 3:**
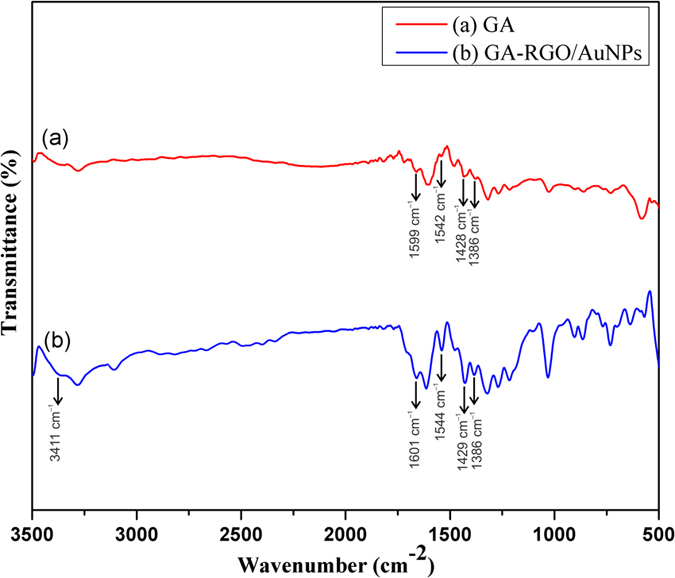
FTIR spectra for (**a**) GA and (**b**) GA-RGO/AuNPs composite.

The prepared composite was further confirmed by FTIR spectroscopy. The functional group of GA radicals are successfully assigned on the surface of RGO/AuNPs, and the results were shown in Fig. 3. As we know that GO exhibits three characteristics peaks assigned at around 3410 cm^−1^, 1395 cm^−1^ and 1062 cm^−1^ for the hydroxyl stretching vibrations of C–OH group, the deformation of OH to C–OH groups and the stretching vibrations of C–O groups, respectively^33^,^34^. After the reduction, it can be seen from GA-RGO/AuNPs (Fig. 3b), the peaks at around 1728 cm^−1^, 1224 cm^−1^ and 1052 cm^−1^ were disappeared and a broad peak assigned at around 3411 cm^−1^, which authenticating that the most of the oxygen functionalities are removed from GO. In addition, the characteristic peaks of GA are assigned at 1542 cm^−1^, 1428 cm^−1^ and 1386 cm^−1^, which are attributed to the C=C stretching and C–H bending vibrations, respectively^21^,^35^. These finding strongly suggest the GA supports on the surface of RGO/AuNPs.

**Figure 4:**
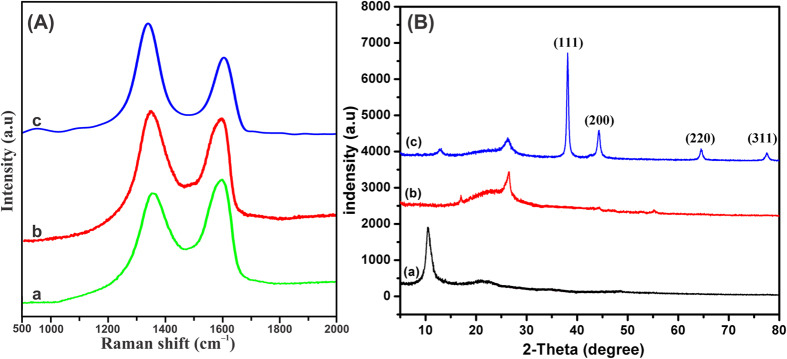
(**A**) Raman spectra and (**B**) XRD patterns for (a) GO, (b) RGO and (c) RGO/AuNPs composites.

The prepared materials were further performed by Raman spectroscopic measurement and the results were shown in Fig. 4A. The Raman spectrum of GO showed two predominant peaks at the wavenumber of 1346.8 cm^−1^ and 1586.3 cm^−1^, which corresponds to the well-documented D and G bands, respectively. The former one arises from the first order scattering of *E*_2g_ phonon of sp^2^ -bonded carbon while the latter is attributed with vacancies, grain boundaries, and amorphous carbon species. After hydrothermal reduction of GO, the D band has become more significant due to the reduction reaction which enhances the defect level and G band has been shifted to lower wave number slightly which indicated that the re-graphitization process^36^. Evidently, the *I*_*D*_*/I*_*G*_ ratio is relatively higher for GA-RGO/Au NPs than the RGO due to the incorporated Au atoms over the surface. To further confirm the phase purity and crystalline nature of the prepared composites, the composite was determined by powder X-ray diffraction (XRD) technique. Figure 4B depicts the XRD patterns of (a) GO, (b) RGO and (c) GA-RGO/AuNPs composites, respectively. The XRD pattern of GO has a dominant peak at the diffraction angle (2θ) of (11.3°) which was attributed to the (001) reflection plane. The calculated inter-planar spacing is to be (0.85 nm) agreed well with the previous report^37^,^38^. After reduction of GO, the 2θ angle of (11.3°) has been completely vanished, and new diffraction peak (Fig. 4B(b)) was appeared at the angle of (26.4°) which was corresponding to the (002) plane. It is suggested that the increased graphitic nature after hydrothermal reduction. As shown in Fig. 4B(c), the four peaks have been observed at the angle of 38.1°, 44.2°, 64.3°, and 77.4° which were assigned to the planes of Au such as (111), (200), (220) and (311) respectively.

**Figure 5:**
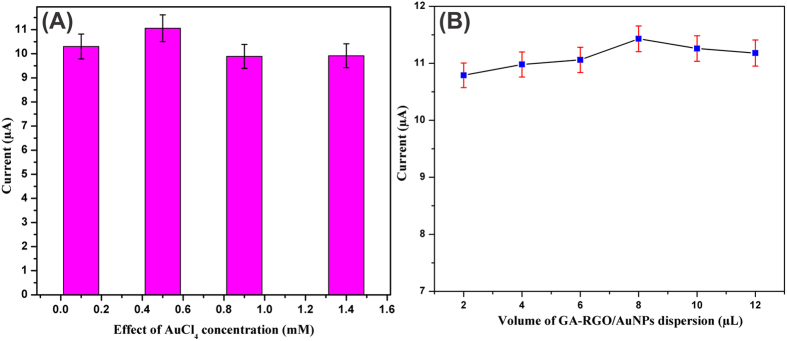
(**A**) Effect of AuNPs and (**B**) influence of the amount of GA-RGO/AuNPs composite towards the detection of DA.

The effect of AuNPs on GA-RGO/AuNPs composite was investigated by CV towards the detection of DA using different concentrations of AuCl_4_ solution, as shown in Fig. 5A. Notice that the enhanced higher oxidation peak current of DA was indentified using 0.5 mM AuCl_4_ in GA-RGO/AuNPs composite. By increasing or decreasing the AuCl_4_ concentrations, the oxidation peak current was significantly decreased which means the growth of Au ions was not uniformly distributed on the RGO surface. The different concentrations of Au ions in GA-RGO/AuNPs composites were characterized by SEM as shown in Fig. S2. It can be seen that 0.5 mM AuCl_4_ solution was successfully anchored and uniformly decorated on the RGO surface, while using the other concentrations of Au ions did not showed uniformity on the RGO surface. Hence, we have used 0.5 mM AuCl_4_ on GA-RGO/AuNPs composite for high performance of electrocatalytic activity and sensing towards DA oxidation. In addition, the influence of amount of GA-RGO/AuNPs on GCE was examined by CV towards the oxidation of DA as shown in Fig. 5B. It can be seen that the response current of DA oxidation (*I*_pa_) was maximum at 8 μL of GA-RGO/AuNPs suspension however the peak current was decreased when increasing or decreasing the concentration. Therefore, 8 μL of GA-RGO/AuNPs was optimized amount for the DA oxidation thus we used this amount for further electrochemical process.

### Electrochemical behavior of GA-RGO/AuNPs toward DA detection

**Figure 6:**
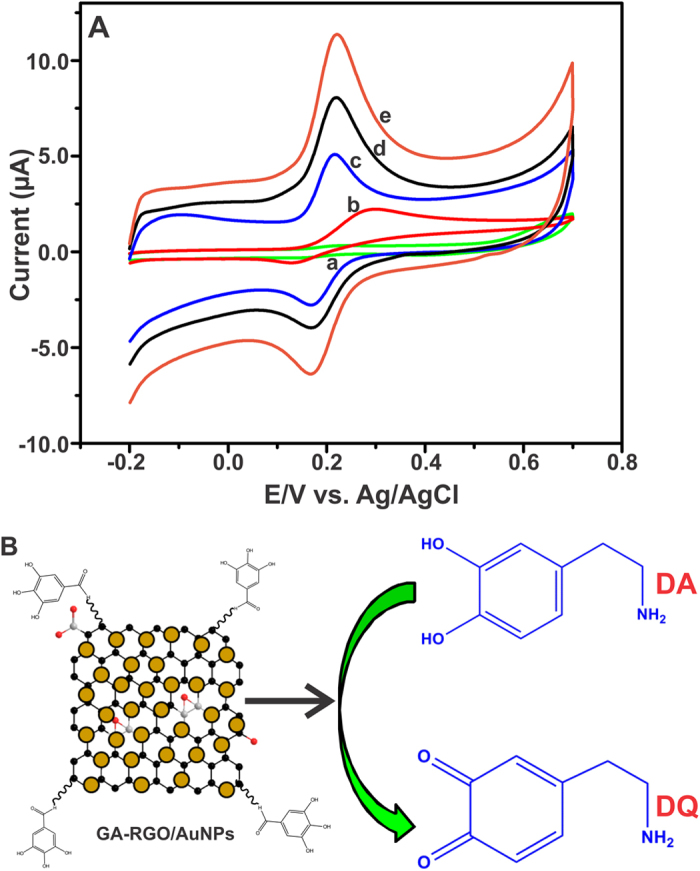
(**A**) CV response for bare (a), GO (b), AuNPs (c), RGO (d) and GA-RGO/AuNPs (e) modified GCEs towards the detection of 100 μM DA, N_2_ saturated 0.05 M PBS, and scan rate of 50 mV/s. (**B**) Plausible sensing mechanism of DA oxidation on GA-RGO/AuNPs.

Cyclic voltammetry (CV) was performed to investigate the electro-oxidation of DA using GA-RGO/AuNPs modified electrode in 0.05 M PBS (pH 7). The CV response of bare (a), GO (b), AuNPs (c), RGO (d) and GA-RGO/AuNPs (e) modified GCEs were studied towards the detection of 100 μM DA in N_2_ saturated 0.05 M PBS at a scan rate of 50 mV/s, was shown in Fig. 6A. Notice that, the bare GCE (without AuNPs) did not shown any obvious response while, the GO modified electrode showed a weak quasi irreversible peak towards DA in the potential window, which suggest the sluggish electrocatalytic process. However, the AuNPs modified electrode showed a good electrochemical redox behavior towards the detection of DA oxidation at 0.218 and 0.167 V, respectively.

On the other hand, the RGO modified electrode displayed a well defined redox behavior towards the oxidation of DA. The anodic and cathodic (*E*_pa_ and *E*_pc_) peak potentials were located at 0.211 and 0.165 V, respectively. Noticeably, the GA-RGO/AuNPs modified electrode showed an enhanced predominant redox behavior and lower potential towards the detection of DA oxidation at 0.206 and 0.158 V for *E*_pa_ and *E*_pc_ with the ∆*E*_p_ of 48 mV, which indicating the higher electrocatalytic activity of GA-RGO/AuNPs. The redox behavior of DA was assigned to the oxidation of DA to dopaminoquinone (DQ) as well as the reduction ascribed as DQ to DA, respectively^29^,^39^. The possible electrochemical pathway for DA oxidation on GA-RGO/AuNPs modified electrode was shown in Fig. 6B. These results conclude that the GA-RGO/AuNPs modified electrode has an enhanced electrocatalytic activity towards the detection DA oxidation.

**Figure 7:**
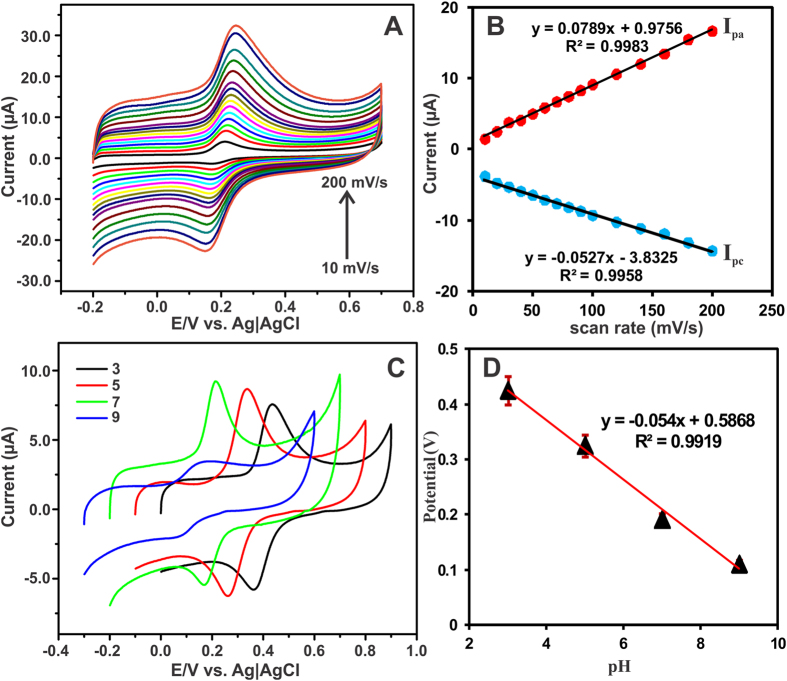
(**A**) CV response for different scan rates from 10–200 mV/s using GA-RGO/AuNPs modified electrode containing 100 μM DA in N_2_ saturated 0.05 M PBS. (**B**) Linear plot for scan rate vs. *I*_pa_ of DA. (**C**) Effect of different pH solutions (3, 5, 7 and 9) using GA-RGO/AuNPs modified electrode containing 100 μM DA, N_2_ saturated 0.05 M PBS, and scan rate of 50 mV/s. (**D**) Calibration plot for *E*_pa_ vs. pH.

The effect of scan rate was investigated towards the oxidation DA using the GA-RGO/AuNPs modified electrode in N_2_ saturated 0.05 M PBS (pH 7) at different scan rates ranging from 10–200 mV/s as shown in Fig. 7A. With increasing the scan rates (10 to 200 mV/s), the oxidation peak current of DA was increased with a systematic redox pairs. Figure 7B showed the linear relationship between the scan rate and the peak current of DA with a correlation coefficient of 0.9983 and 0.9958 for anodic (*I*_pa_) and cathodic (*I*_pc_) peak currents, respectively, which suggest that the electro-oxidation of DA was controlled by adsorption–controlled electrochemical process on the electrode surface^40^,^41^,^42^. In addition, the protonation of DA oxidation at different pH solutions using GA-RGO/AuNPs modified electrode was studied by CV and the results are shown in Fig. 7C. It can be seen that the higher oxidation peak current was observed in pH 7, while increasing or decreasing the solution pH (3, 5 and 9) peak current was gradually decreased and the peak potential was shift towards positive and negative directions. Hence, the pH 7 is an optimum for the electro-oxidation of DA at GA-RGO/AuNPs modified GCE. In addition, Fig. 7D shows a linear relationship between the *E*_pa_ and the pH (3, 5, 7 and 9) solution. The slope value was assigned to be 54 mV/pH, which is nearly close to previously reported DA sensor. Therefore, the electro-oxidation of DA was described by equal number (n = 2) of proton and electron transfer reaction on the electrode surface^43^,^44^,^45^.

### Determination of DA

**Figure 8:**
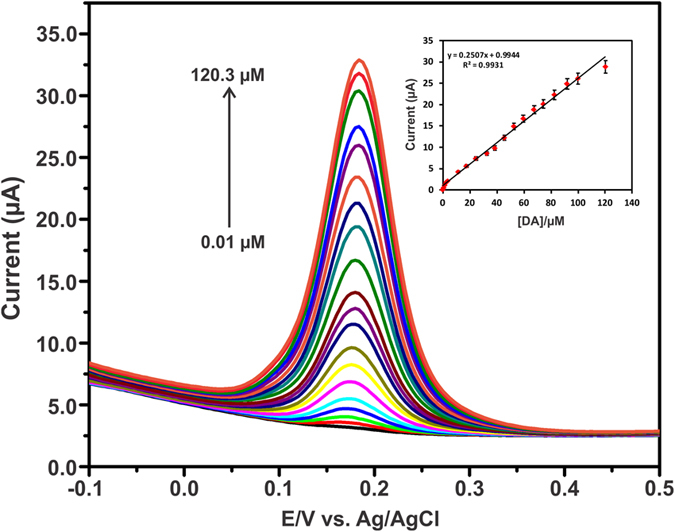
DPV response for GA-RGO/AuNPs modified electrode upon the successive additions of 0.01–120.3 μM DA in N_2_ saturated 0.05 M PBS. Inset: Calibration plot of DA concentrations vs. *I*_pa_.

In order to evaluate the efficiency of the sensor, DPV was performed for the determination of DA using GA-RGO/AuNPs modified electrode in N_2_ saturated 0.05 M PBS. Figure 8 depicts the DPV response of DA oxidation at GA-RGO/AuNPs modified electrode for the successive additions of DA (0.01–120.3 μM) in N_2_ saturated 0.05 M PBS. It can be seen that the oxidation peak current was significantly increased with increasing the concentrations of DA ranging from 0.01–120.3 μM. The oxidation of DA to DQ, the anodic peak current was liner dependant over the concentrations of 0.01–100.3 μM as shown in Fig. 8(inset). The linear regression equation is expressed as *I*_pa_ (*μ*A) = 0.2507 [DA] (*μ*mol L^−1^) + 0.9944 (R^2^ = 0.9931). The calculated limit of detection (LOD) was achieved to be 2.6 nM based on signal to noise ratio (*S/N = 3*) with a sensitivity of 3.58 μA μM^−1^ cm^−2^. The advantage of GA-RGO/AuNPs modified electrode and the analytical performance of the sensor (linear range, LOD and sensitivity) were compared with previously reported DA sensors and the results were summarized in Table 1** **46,47. These findings concluded that the GA-RGO/AuNPs modified electrode is a suitable sensing platform for DA with excellent analytical performance.

### Abbreviations

GNF: Gold nanofilm; AuNPs: Gold nanoparticles; PANI: Polyaniline; PE: Polyelectrolyte; PS: Polystyrene; BBD: Boron-doped diamond; GO: Graphene oxide; Pt: Platinum; MWNT: Multiwalled carbon nanotubes; β-CD: β-cyclodextrin; GR: Graphene; MnO_2_: Manganese dioxide; Fe_3_O_4_: Iron oxide; GNs: Graphene nanospheres; RGO: Reduced graphite oxide.

### Selectivity, stability and reproducibility

**Figure 9:**
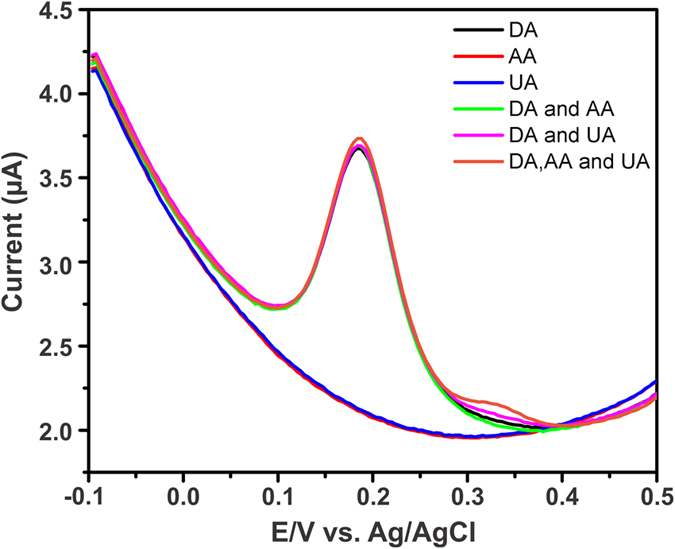
DPV response of GA-RGO/AuNPs modified electrode in presence of 5 μM DA and 50 fold excess concentrations of AA and UA in N_2_ saturated 0.05 M PBS (pH 7).

It is well known that the ascorbic acid (AA) and uric acid (UA) are two potential biological molecules commonly interfering with DA detection. Hence, we have investigated the selectivity of DA sensor in the presence of AA and UA by using DPV and shown in Fig. 9. It can be notice that, the AA and UA did not show any obvious signal even in the presence of 50 fold higher concentrations. Meanwhile, the AA and UA were added in presence of DA, there is no change in the peak potential and peak current of DA. Hence, the proposed GA-RGO/AuNPs modified electrode was more suitable for selective and high performance of DA sensor in presence of AA and UA. In addition, we have also performed the selectivity of the sensor in presence of common metal ions and the results were shown in Fig. S3A. It can be seen that 200 fold higher concentrations of the metal ions did not show any apparent signal in the potential window. Meanwhile, the response time of the sensor was calculated by amperometry method. The sensor was reached the steady state current within 3 s and the result was shown in Fig. S3B. These findings are further strongly suggested that the proposed GA-RGO/AuNPs modified electrode was used for fast response, highly selective and sensitive detection of DA in presence of AA and UA. The stability of GA-RGO/AuNPs composite was studied by CV. The storage stability of GA-RGO/AuNPs modified electrode was investigated carefully and the oxidation peak current of DA was monitored by CV. Prior to analysis, The GA-RGO/AuNPs modified electrode retains 96.5% of its initial response after 15 days time interval with the relative standard deviation (RSD) of 3.42%. The monitored response current of DA for every 3 days was shown in Fig. S4A. The reproducibility of the sensor was examined by CV. Independently prepared five different GA-RGO/AuNPs modified electrode was evaluated in a solution containing 100 μM DA, N_2_ saturated 0.05 M PBS, and scan rate of 50 mV/s. The response current was shown in Fig. S4B. The calculated RSD value of the sensor was 2.68%. This result suggests that good storage stability and repeatability of the proposed sensor.

### Real sample analysis

The newly constructed GA-RGO/AuNPs modified electrode was further applied for the determination DA in human blood serum and urine samples *via* standard addition method. The real samples were diluted with 0.05 M PBS (pH 7) before the analysis. Briefly, the human serum and urine samples were 10 times diluted with 0.05 M PBS and spiked with 0.1 mM DA. These samples are used for the analysis of DA and without DA spiked samples are used as a control. The observed response current of DA for human serum and urine samples were shown in Fig. S5. The recovery values of human serum and urine samples were summarized in Table 2. It can be noted that the proposed sensor retains the recovery ranges from 95.2–98.6%. This finding clearly suggested that the GA-RGO/AuNPs modified electrode has suitable for the determination of DA in human serum and urine with appropriate recoveries for practical applications.

## Conclusions

In summary, we have demonstrated a highly efficient GA supported reduced graphene oxide/gold nanoparticles (GA-RGO/AuNPs) for selective and sensitive detection of DA sensor. The as-prepared GA-RGO/AuNPs modified electrode displayed an excellent electrocatalytic activity and achieved lower LOD, wide linear response, higher stability and reproducibility towards the detection of DA. This electrode was further employed for the detection of DA in presence of AA, UA and common metal ions for the selectivity of the sensor. Moreover, the GA-RGO/AuNPs modified electrode successfully applied for the determination of DA in human serum and urine samples with satisfactory recoveries for practical analysis. Considering the simplicity, higher stability and excellent analytical performance, we believe that the proposed electrode material could be more suitable for biosensor and electronic device based senor applications.

## Experimental

### Chemicals

Pristine graphite (diameter < 20 μm) and dopamine were purchased from Sigma-Aldrich. Gold (III) chloride trihydrate (HAuCl_4_.3H_2_O, 99.9%) and gallic acid (C_7_H_6_O_5_), Na_2_HPO_4_, NaH_2_PO_4_, H_2_SO_4_ and NaOH were supplied from Sigma-Aldrich. Human blood serum sample was collected from valley biomedical, Taiwan product & services, Inc. Urine samples were collected from Taipei Tech student with their permission and dilute the sample for real sample analysis. The supporting electrolyte phosphate buffer solution (0.05 M PBS, pH 7) was prepared by dispersing 0.05 M Na_2_HPO_4_ and NaH_2_PO_4_ solutions and the other pH solutions were prepared using either H_2_SO_4_ or NaOH. All chemicals used were analytical grade and without further purification.

### Instruments and apparatus

The Raman spectrum was carried out using Dong Woo 500i, Korea equipped with a 50× objective and a charge coupled detector. X-ray diffraction (XRD) patterns were recorded from XPERTPRO (PANalytical B.V., The Netherlands) diffractometer (Cu Kα radiation, k = 1.54 Å). The lattice size of the composite was examined by high resolution transmission electron microscope (FE-TEM, JEM-3000 F, JEOL operated at 300 kV with a point to point resolution of 0.17 nm). The surface morphologies of composites were characterized by using scanning electron microscope (SEM) using HitachiS-3000H scanning electron microscope and the elemental mapping was performed by HORIBA EMAX X-ACT attached with HitachiS-3000H microscope and high resolution transmission electron microscopy (HR-TEM, JEOL, JEM-3000F) operated at 300 kV. All electrochemical measurements were carried out using a computerized CHI-1205B, CHI-750 electrochemical work stations. A three-electrode system consisting of glassy carbon electrode (GCE) used as the working electrode, platinum wire (0.5 mm) and an Ag/AgCl electrode (Sat. KCl) used as counter and reference electrodes. All electrochemical experiment was carried out an inert atmosphere (N_2_ atmosphere) at room temperature. The modified electrode was stored in 4 °C, when not in use.

### Synthesis and fabrication of GA-RGO/AuNPs composite

Graphite oxide (GO) was synthesized from pristine graphite using modified Hummer’s method^48^. To synthesize the GA-RGO/AuNPs composite, first 12.5 mg of as synthesized GO was dispersed in 25 mL of deionized water (DI water) under a magnetic stirring for 1 h at room temperature. After that the HAuCl_4_.3H_2_O (0.5 mM) was added to the aqueous GO dispersion. The suspension was keeping at room temperature for 30 min until magnetic stirring to support the interaction of Au ions with the graphene surface. Then, NH_3_.H_2_O was added to the solution to adjust the pH value to upon 8. After completing the process, C_7_H_6_O_5_ (2 mg/mL) was added to the suspension under magnetic stirring for 30 min. Next, the solution was heated to 60 °C for 2 h, the color changes from light brown to dark golden brown, suggesting the formation of RGO and AuNPs with the aid of GA. The reaction mass was cooled and was washed with deionized water by centrifugation (5000 RPM, for 30 min) to remove the unreacted AuNPs and other particles. The GA-RGO/AuNPs composite was collected and dry at 60 °C for 1 h and used for further studies. Before to use the GA-RGO/AuNPs modified electrode, GCE was polished with alumina slurry and thoroughly washed with DI water, followed by ethanol by using ultra sonication. The mirror like GCE was dried at room temperature. About 8 μL of the aqueous dispersion of as-prepared GA-RGO/AuNPs composite was drop cast on the electrode surface. The modified electrode was dried at ambient condition in inert atmosphere. The obtained GA-RGO/AuNPs modified electrode was used for further electrochemical experiments.

## Additional Information

**How to cite this article**: Thirumalraj, B. *et al*. One-Pot Green Synthesis of Graphene Nanosheets Encapsulated Gold Nanoparticles for Sensitive and Selective Detection of Dopamine. *Sci. Rep.* **7**, 41213; doi: 10.1038/srep41213 (2017).

**Publisher's note:** Springer Nature remains neutral with regard to jurisdictional claims in published maps and institutional affiliations.

## Supplementary Material

Supplementary Information

## Figures and Tables

**Figure 1:**
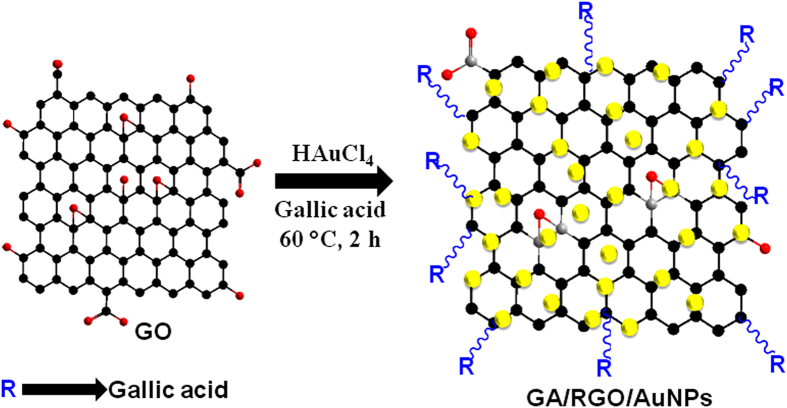
Schematic representation of the preparation of GA-RGO/AuNPs composite.

**Table 1:** Comparison of the analytical performances of GA-RGO/AuNPs modified electrode with previous reported AuNPs based DA sensor.

Modified electrode	Detection Method	LOD (μM)	Linear range (μM)	Sensitivity	Refs
GNF electrode	DPV	1.5	1.5–27.5	—	48
Nanostructured Au	Amperometry	0.026	0.2–600	0.178 μA μM^−1^ cm^−2^	49
AuNPs/PANI	Amperometry	0.8	3–115	—	50
Au/PE/PS/BBD	CV	0.8	5–100	—	51
GO	DPV	0.27	1–15	—	52
Graphene/AuNPs	DPV	1.86	5–1000	510.2 μA mM^−1^ cm^−2^	53
Pt@Au/MWNT	Amperometry	0.08	Up to 120	1.16 mA mM^−1^ cm^−2^	54
Au nanowire	Amperometry	0.4	0.4–250	—	55
AuNPs/β-CD/GR	DPV	0.15	0.5–150	—	56
GO-WCNT/MnO_2_/AuNP	Amperometry	0.17	0.5–2500	233.4 μA mM^−1^ cm^−2^	57
Fe_3_O_4_@GNs/Nafion	DPV	0.007	0.02–130	—	58
AuNPs@PS/RGO	DPV	0.005	0.05–20	3.44 μA μM^−1^	47
GA-RGO/AuNPs	DPV	0.0026	0.01–100.3	3.58 μA μM^−1^ cm^−2^	This work

**Table 2:** Determination of DA in human serum and urine samples by using GA-RGO/AuNPs modified electrode.

Sample	Spiked (μM)	Found (μM)	Recovery (%)	RSD (%)
Human serum	5.0	4.76	95.2	2.4
	5.0	4.89	97.8	2.7
Urine sample	5.0	4.84	96.8	3.1
	5.0	4.93	98.6	3.3

The relative standard deviation (RSD) is related for n = 3.
