# Supplementary material for: One-Pot Green Synthesis of Graphene Nanosheets Encapsulated Gold Nanoparticles for Sensitive and Selective Detection of Dopamine
Source: Sci Rep. 2017 Jan 27;7:41213. doi: 10.1038/srep41213 (PMC5269579; doi:10.1038/srep41213)
Supplement: Supplementary Information [file srep41213-s1.pdf]

Supporting information

One-Pot Green Synthesis of Graphene Nanosheets Encapsulated Gold Nanoparticles for Sensitive and Selective Detection of Dopamine

Balamurugan Thirumalraj,[a] Chellakannu Rajkumar,[a] Shen-Ming Chen*[a] and Selvakumar Palanisamy [a]

[a]Department of Chemical Engineering and Biotechnology, National Taipei University of Technology, Taipei 106, Taiwan, ROC.

* Corresponding author: S.M. Chen; E-mail: smchen78@ms15.hinet.net; Fax: +886 2270 25238; Tel: +886 2270 17147.

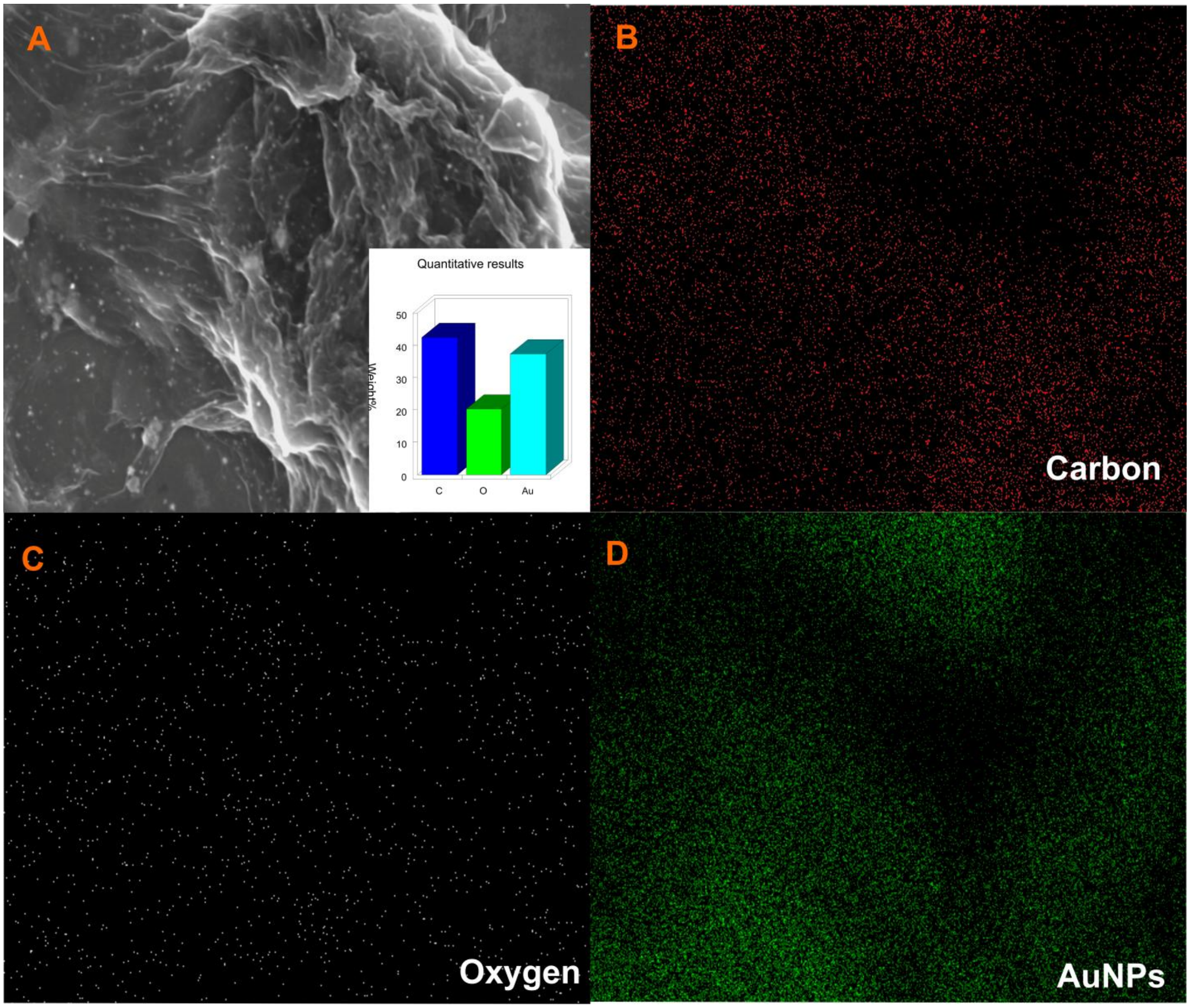

Figure S1. SEM image for GA-RGO/AuNPs (inset: Elemental weight percentage for C, O and Au) and corresponding elemental mapping (B–D).

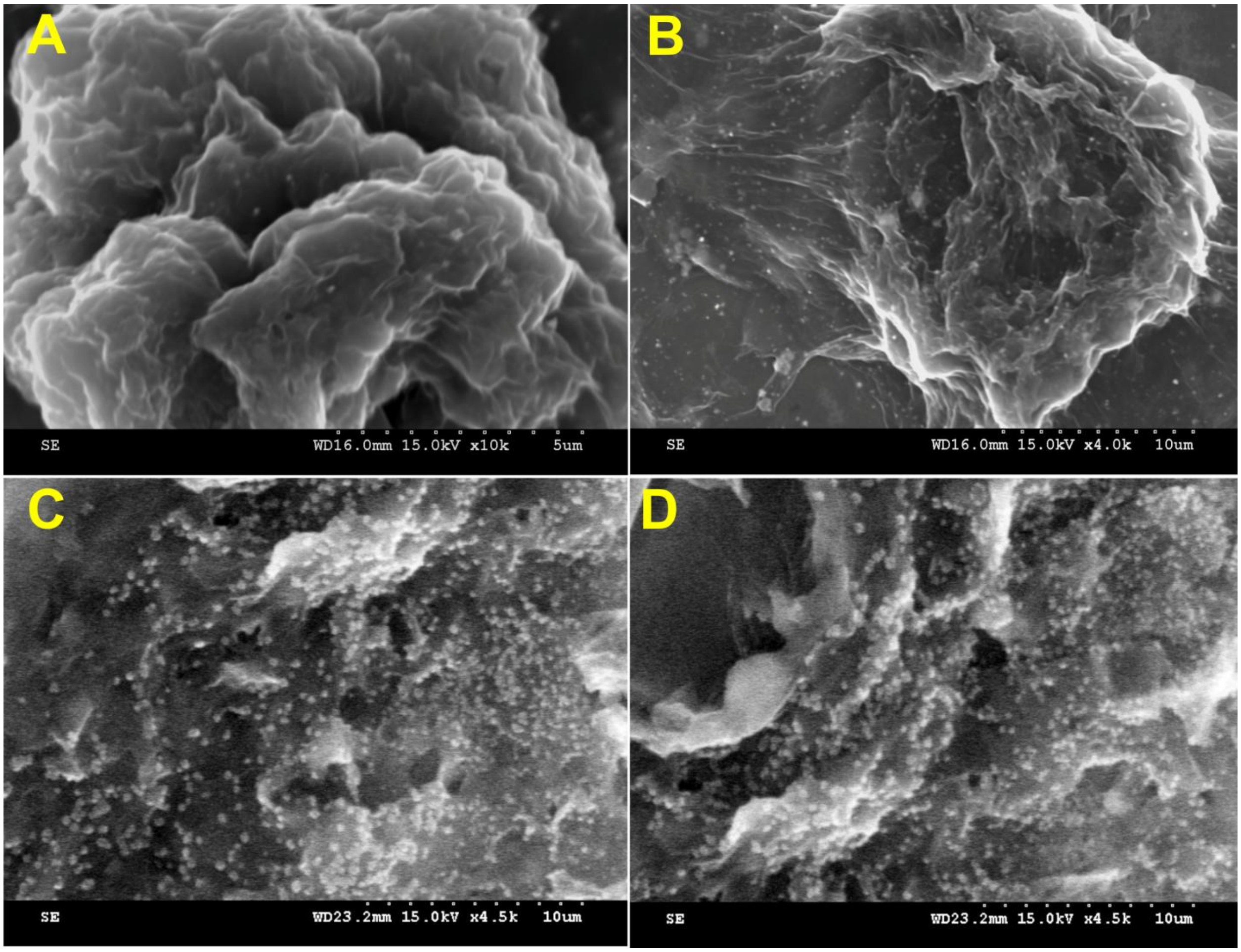

Figure S2. SEM images of GA-RGO/AuNPs composites in different concentrations of $AuCl_4$ solution 0.1 mM (A), 0.5 mM (B), 0.7 mM (C) and 1.4 mM (D).

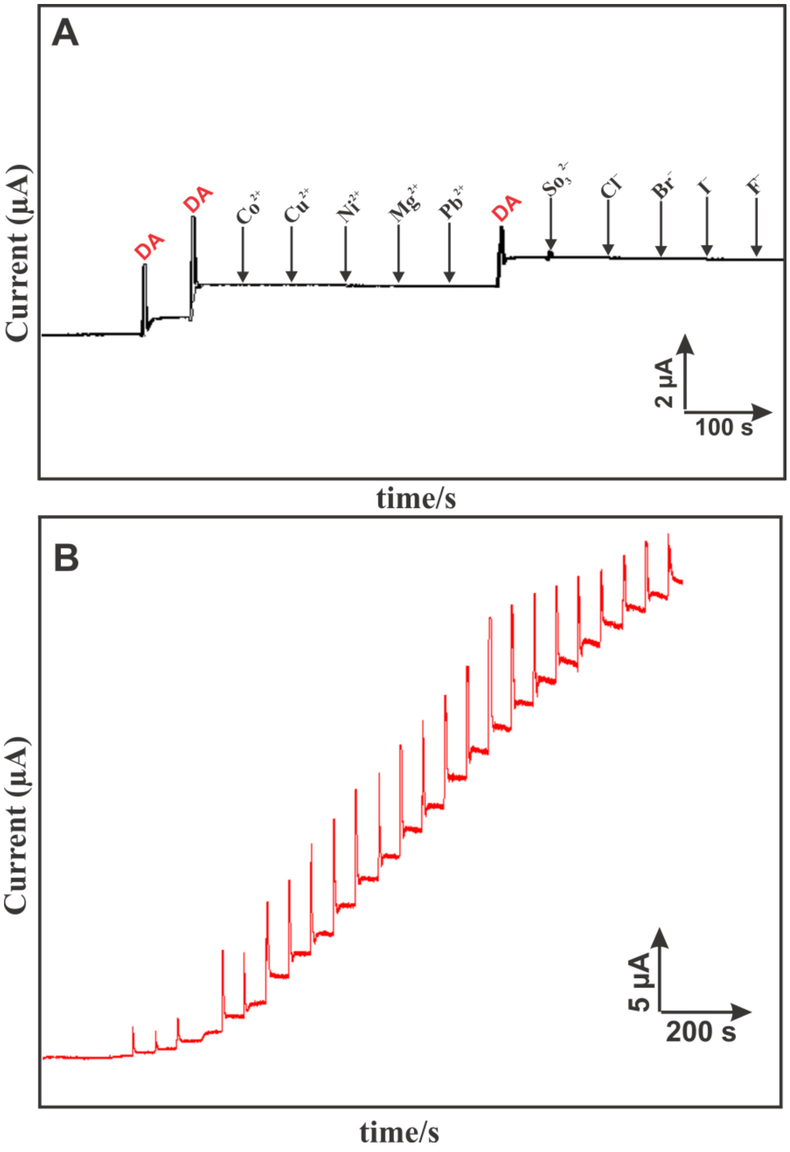

Figure S3. (A) Amperometric i-t responses for the successive addition of 1 μM DA and 200 fold excess concentrations of Co^{2+}, Cu^{2+}, Ni^{2+}, Mg^{2+}, Pb^{2+}, SO_3^{2-}, Cl^-, Br^-, I^- and F^- in 0.05 M PBS (pH 7.0) solution. (B) Amperometric i-t response for the response current for DA using GA-RGO/AuNPs. The response time of the sensor = 3 s.

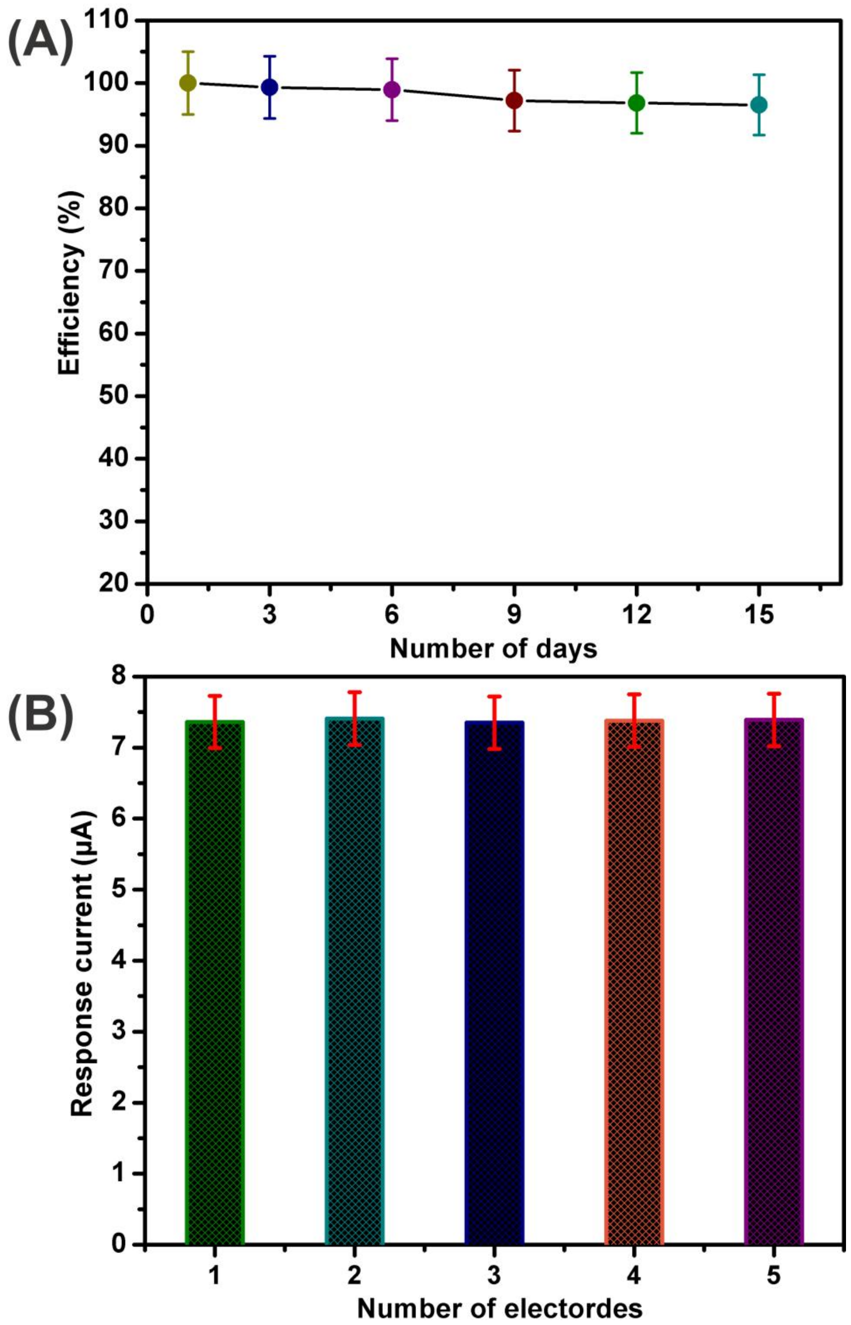

Figure S4. (B) Responses current for DA oxidation using GA-RGO/AuNPs modified electrode for every 3 days time intervals. (B) Response current for DA oxidation using five different GA-RGO/AuNPs in solution containing 100 μM DA, N_2 saturated 0.05 M PBS, and scan rate of 50 mV/s.

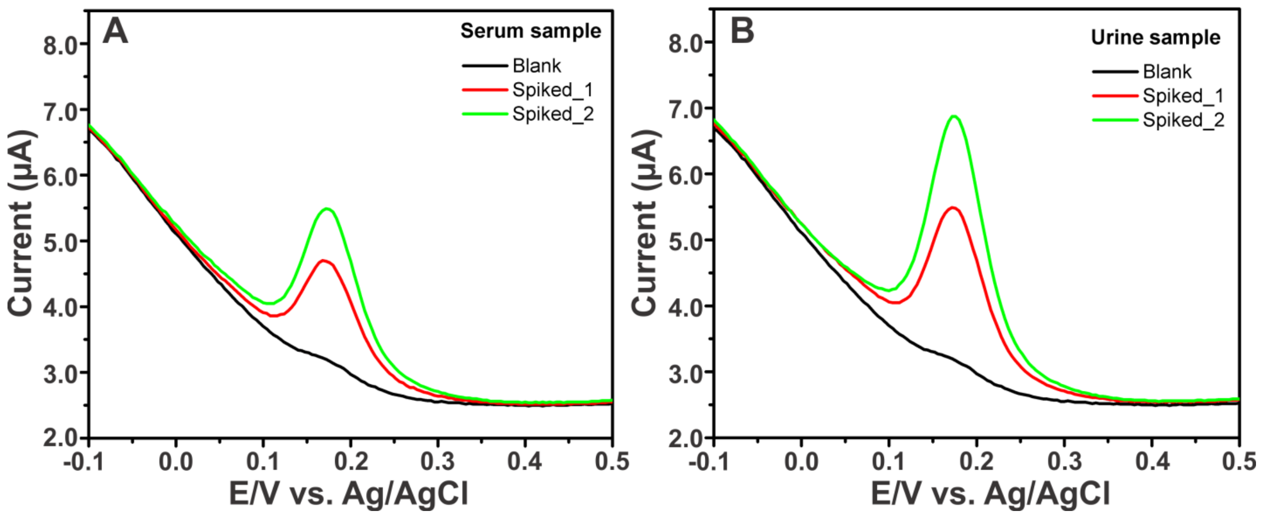

Figure S5. (A) DPV response for the determination of DA in human serum sample and (B) DPV responses for the determination of DA in urine sample. Experimental condition: 0.05 M PBS (pH 7), N_2 atmosphere.
